# Frasera Carolinensis—American Columbo

**Published:** 1860-10

**Authors:** 


### Frasera Carolinensis—American Columbo.

This indigenous plant, found in most of the Southern and South-western States, may be used in many instances with equal benefit to that derived from *Coculus Palmatus—Columbo*. The Medicinal Garden here, was favored by a friend in Walker Co., Ga., with a root of this plant, but owing to the lateness of the Season when it was obtained, never sprouted. We hope to succeed in getting another trial at the culture of Frasera.

Charles A. Lee, M. D., in one of his articles for the *Journal of Materia Medica*, on "*Indigenous Tonics*," says:

"The American Columbo is one of our handsomest native plants, and the only one of its genus as yet discovered. It flourishes in the southern and western portions of the United States, especially in Arkansas and Missouri. In the western glades of Kentucky, Raffinesque states that he has seen it ten feet high, with a pyramid of crowded blossoms, four or five feet long. It is properly a triennial, though some botanists consider it a biennial; its stem and flowers only being produced in the third year, the radical leaves being the only part of the plant which previously appear above ground, and flowering from May to July. The root is to be collected in the spring of the third year, or the autumn of the second, cut into transverse slices like columbo, and dried.

The root of the Frasera is triennial, very large, weighing several pounds, yellow, rugose, spindle shaped and horizontal; the whole plant smooth, from five to ten feet high, cylidrical, and solid. The *leaves* are sessile, verticillate, entire, the radical elliptical and obtuse, long; cauline ones smaller and narrower. The *flowers* are yellowish white, numerous, and form a large pyramidal panicle. The plant seems to have been first discovered by William Bartram, and dedicated to John Fraser, a well-known and indefatigable collector in this country towards the close of the last century. It is called *American Columbo*, from the resemblance of the root when cut into transverse slices and dried, to the foreign columbo; also, from the similarity of its medical properties. When sliced horizontally, the root closely resembles that of gentian, to which it is botanically allied, belonging to the same botanical family."

Again, Dr. Lee says, under the head of the "*Therapeutical properties and Effects*" of American Columbo:

"*Therapeutical Properties and Effects.*—"The root when freshly dug is both emetic and cathartic, in this respect resembling most of our indigenous tonics. When carefully dried, it is a mild tonic, fulfilling the same indications as the other simple bitters. According to some writers, it combines laxative with its tonic properties, in this respect resembling rhubarb, while its sweetish bitter taste reminds us of gentian. We have, however, never observed any laxative effects from its use, except in the fresh state. In atonic affections of the digestive organs, the cold infusion proves a serviceable remedy. Raffinesque states that its internal and external use has cured extensive gangrene of the lower extremities, when bark had failed; also, that it proves successful in intermitents, and is greatly used in the western States in fevers, colics, griping, nausea, diarrhea, &c. An infusion of the fresh root is often used as a substitute for rhubarb, in diseases of children, as well as to obviate the constipation incident to pregnancy. It has the advantage of not stimulating; cold water is said to add to its efficiency, and prevent nausea or emesis. A teaspoonful of the powdered fresh root, in hot sweetened water, acts as a very prompt emetic. Clayton & Schœepf speak of its having been succesfully employed in jaudice, scurvy, gout, suppressed menstruation, and even hydrophobia. In many respects it seems to resemble the menyanthes, (*buckbean*) and fulfils the indications pointed out under that plant.

*Preparations and Administration.*—The frasera may be given in powder, infusions, fluid extract, tincture, compound tincture, syrup, and fraserin.

The *powder* may be given in a dose of from thirty grains to one dram; although it is not an eligible form.

The *infusion* is made in the proportion of an ounce of the bruised root to one pint of boiling water, of which the dose is from one to two fluid ounces, repeated several times a day.

The *fluid extract*, not yet introduced, would be the most eligible form, of which one to two drams would constitute a dose.

The *tincture* is chiefly valuable as a stomachic tonic, in doses of one dram.

The *compound tincture*, as a tonic, laxative, and carminative, may be prepared from the root of frasera, rhubarb, and gentian, each one ounce; coriander, fennel and dill seed, each two drams; cardamom, half an ounce; raisins, half a pound; diluted alcohol, three pints. Macerate for fourteen days, express, and filter through paper. Dose, one to three drams.

*Fraserin* is the peculiar principle, with the resin combined. Dose, from two to four grains.

The *frasera* deserves to be cultivated as an ornamental plant. There is no good reason why the *cocculus palmatus* (columbo,) should not be grown in our Southern States, where both climate and soil are well adapted to its successful cultivation. Both may be propagated alike from the seed and root."

